# High-Performance Size-Based Microdevice for the Detection Of Circulating Tumor Cells from Peripheral Blood in Rectal Cancer Patients

**DOI:** 10.1371/journal.pone.0075865

**Published:** 2013-09-16

**Authors:** Wenjie Sun, Chunping Jia, Ting Huang, Weiqi Sheng, Guichao Li, Honglian Zhang, Fengxiang Jing, Qinghui Jin, Jianlong Zhao, Gang Li, Zhen Zhang

**Affiliations:** 1 Department of Radiation Oncology, Fudan University Shanghai Cancer Center, Shanghai, China; 2 Department of Oncology, Shanghai Medical College, Fudan University, Shanghai, China; 3 Key Laboratories of Transducer Technology and Science and Technology on Micro-system Laboratory, Shanghai Institute of Microsystem and Information Technology, Chinese Academy of Sciences, Shanghai, China; 4 Department of tumor chemotherapy, the Affiliated Hospital of Nantong University, Jiangsu, China; 5 Department of Pathology, Fudan University Shanghai Cancer Center, Shanghai, China; Centro Nacional de Investigaciones Oncológicas (CNIO), Spain

## Abstract

Since individualized therapy becomes more and more important in the treatment of rectal cancer, an accurate and effective approach should be established in the clinical settings to help physicians to make their decisions. Circulating tumor cells (CTCs), originated from either primary or metastatic cancer, could provide important information for diagnosis and monitoring of cancer. However, the implication and development of CTCs are limited due to the extreme rarity of these tumor cells. In this study we fabricated a simple and high-performance microfluidic device, which exploited numerous filtered microchannels in it to enrich the large-sized target tumor cells from whole blood. A very high CTC capture efficiency (average recovery rate: 94%) was obtained in this device at the optimum flow rate of 0.5 mL/h and channel height of 5 µm. Additionally, we used this device for detecting CTCs in 60 patients with rectal cancer. The CTC counts of rectal cancer patients were significantly higher than those in healthy subjects. Furthermore, the CTC counts detected by this device were significantly higher than those by EpCAM bead-based method for rectal cancer patients with various stage. Especially, for localized rectal cancer patients, the positive rates of samples with more than 3 CTCs per 5 mL blood by use of microdevice vs. EpCAM-based ones were 100% vs. 47%, respectively. Thus, this device provides a new and effective tool for accurate identification and measurement of CTCs in patients with rectal cancer, and has broad potential in clinical practice.

## Introduction

In recent years, the incidence of rectal cancer is dramatically increasing all over the world [1]. Despite of the recent advances in diagnostic and treatment techniques, the outcome after treatment remains poor mainly because of the appearance of the distant metastases [2]–[4].

Circulating tumor cells (CTCs), shed from either primary or metastatic cancer, have been identified in the peripheral blood of patients and are often associated with cancer recurrence and metastasis [5]–[6]. Consequently, the detection of CTCs would be expected to provide a powerful tool for cancer prognosis, diagnosis of minimal residual disease, assessment of tumor sensitivity to anti-cancer drugs, and application of individualized treatment [7].

However, attempts to study CTCs are limited by their rarity, with concentrations as low as one CTC per billion circulating hematologic cells [8]. Thus CTCs must be enriched, isolated and properly identified in order to be clinically useful. Currently, one of the most commonly used methods of CTC detection is based upon the use of magnetic bead conjugated antibodies against epithelial tumor related surface antigens such as epithelial cell adhesion molecule (EpCAM) [9]–[10]. The CellSearch (Veridex, NJ, USA) system is a typical example using anti-EpCAM magnetic bead-based method to detect CTCs and it's the only system approved by the US FDA for clinical utilization in metastatic breast, colorectal and prostate cancer till now [11]. Nevertheless, some data suggested that epithelial antigen might be lost on CTCs due to the epithelial-mesenchymal transition (EMT), which was considered to be a crucial event in metastatic process [12]–[13]. This may be the main problem that restricts the wide application of EpCAM-based method in clinical practice.

On the other hand, another commonly used approach to separate CTCs from blood samples is based on differences in cell size and deformability between CTCs and hematologic cells. In general, the sizes of CTCs range from 15 to 25 µm in diameter, larger than those of hematologic cells (Average sizes of erythrocytes and peripheral blood lymphocytes are 8 µm and 7–10 µm in diameter, respectively.) [14]–[15]. ISET(Les Ulis, France), which enumerates CTCs by blood filtration through polycarbonate Track-Etch-type membranes with 8 µm cylindrical pores, is a representative one with comparatively high capture efficiency [16]. However, the pores of polycarbonate filters, which are fabricated by track etching, are randomly placed with nonuniform density [17]–[18]. Recently, the development of microfabrication technique has allowed the introduction of microfluidic approaches for capturing these rare cells, which might make up the weakness of ISET [19]–[20]. Using one-layer parylene-C membrane micro-filters, Zheng S et al. [21] recovered 89.5%±9.5% human prostate cancer cells that were spiked into whole blood of healthy donors. In addition, Tan and Lim et al. [14] developed a microfluidic device with multiple arrays of crescent-shaped wells and obtained capture efficiencies higher than 80% for breast and colon cancer cell lines. Furthermore, Bhagat et al. [22] utilized a rectangular micro-channels patterned with a contraction-expansion array and acquired the recovery rate higher than 90% for human breast cancer cells of MCF-7. Nevertheless, these methods only detected the samples with cancer cell lines spiked into healthy blood, but lacking the verification of CTCs in the blood of cancer patients as well as the further analysis of their clinical application.

New therapeutic strategies improving the efficacy against metastatic disease and accurate biomarkers for the follow-up of rectal cancer patients are the leading challenges, together with early detection and screening in high-risk populations. In this study, we designed and fabricated a microfluidic filtration device to capture these rare CTCs based on the differences in size between tumor cells and blood constituents. In our device, blood flow passes through a micro-channel-based system allowing size-selective isolation of rare tumor cells, with very high capture efficiency and fully repeatability. After immunofluorescence staining, CTCs can then be identified and enumerated directly under fluorescence microscope. Our device is a highly effective tool for the capture of rare CTCs without the requirement of large and expensive apparatus. The purposes of this study were as follows: Firstly, we measured and optimized the parameters of our microfluidic device by spiking colorectal cancer cells into blood samples. Secondly, we enumerated and analyzed the CTC capture efficiency both in spiked cells model and in blood samples from patients with rectal cancer. Lastly, we compared our device with anti-EpCAM antibodies conjugated immunobead-based method.

## Materials and Methods

### Device Design and Fabrication

**Figure 1:**
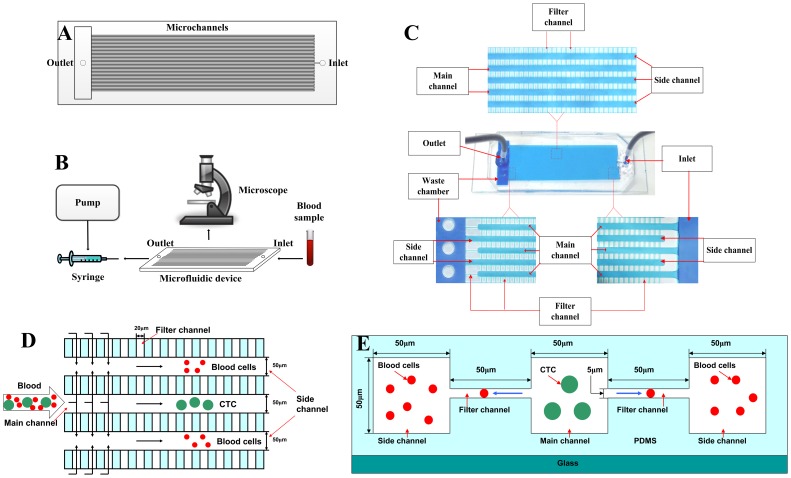
The theory and structure of the microdevice. A) The schematic of the microfluidic device; B) The schematic of workstation setup for CTC isolation; C) The structure of the device under the microscope; D) The schematic showing the theory of the device in vertical view; E) The schematic showing the theory of the device in profile view.

As shown in Figure 1, the microfluidic device consists of 80 main channels and 81 side channels, which are arranged in an interdigital manner to minimize the chip footprint. A group of narrow parallel-arranged filter channels are designed to connect each pair of main channel and adjacent side channel. Both main channels and side channels have the cross sections of 50 µm in width and 50 µm in height, while those of filter channels have widths of 20 µm and heights that varied from 2 µm to 8 µm. For each main channel, the right side is connected directly with the sample inlet, and the left side is linked via a filter channel to a waste chamber, with an array of micro-posts that are designed to prevent chamber collapse. For each side channel, the right side is blind and the left one is connected directly with waste chamber. After the blood sample is loaded at the inlet, a negative pressure is applied to the outlet, which aspirates the blood sample into the main channels. Meanwhile, due to pressure difference between the main channel and the side channel, most of the small-sized hematologic cells in whole blood such as erythrocytes and leukocytes can be filtered into their adjacent side channels via filter channels and then eliminated from the waste chamber. The large-sized cells such as tumor cells cannot pass through the narrow filter channels and then remain in the main channels. This is the theory of the isolation of CTCs for this device.

Our device was fabricated by bonding a hybrid polydimethylsiloxane (PDMS) slab containing three-dimensional micro-channels with a glass slide. The microfluidic device was fabricated through the well-established multi-layer soft lithography process [23]. Briefly, a two-level master was prepared from a negative photo-resist, SU-8 (Microchem, USA). Subsequently, degassed PDMS (mixed in a 10∶1 ratio of PDMS base with curing agent, Sylgard 184, Dow Corning Inc., USA) was cast over the master mold and baked at 90°C for 1 h in an oven. After curing, the PDMS slab was carefully peeled off from the master mold. One inlet and one outlet were punched through the PDMS using a needle with a flattened tip. The PDMS slab was then bonded with a glass slide after oxygen plasma treatment, and the process was described as follows: The oxygen plasma bonding was done using dedicated plasma cleaner (PDC-32G-2, Harrick Plasma Corp., USA). The glass slide and PDMS slab were placed in the chamber of plasma cleaner for 50 seconds. After that, PDMS slab and glass slide were taken out of the chamber, and PDMS was placed and pressed on the glass immediately.

### Cell Culture and Cell Spiking

The human colorectal cell line HT-29 that was obtained from cell bank of American Type Culture Collection was cultured in McCoy's 5A medium (Gibco, Carlsbad, CA, USA) supplemented with 10% fetal bovine serum (Gibco, Carlsbad, CA, USA). Cell culture was carried out in an incubator at 37°C in 5% CO_2_. Growth medium was aspirated and cells were subsequently harvested using 0.25% trypsin. Immediately before experiments, cells were rinsed with PBS buffer and resuspended. By following the manufacturer's instructions, the cells were treated with Vybrant DiI cell-labeling solutions (Carlsbad, Invitrogen, CA) for 5 min at 37°C, then rinsed with PBS buffer and resuspended. The concentration of cells was determined by counting with a hemacytometer. The desired concentration of cells was prepared by diluting cell suspensions in PBS buffer. Labeled cells with known numbers were spiked into blood samples for further measurements.

### Blood Specimen Collection and Processing

The blood samples from 60 patients with rectal cancer were obtained from March to December of 2012. All patients were confirmed pathologically as rectal carcinoma by biopsy or surgery. Of these, 30, 10 and 20 patients were confirmed as newly diagnosed Stage II-III, local recurrence and distant metastasis, respectively. For all these 60 patients, 10 mL blood samples were obtained for CTC detection, including 5 mL by microfluidic device and 5 mL by EpCAM-based method.

As a control group, blood samples were also drawn from 30 healthy volunteers who had no known illness or fever at the time of draw and no history of malignant disease.

All specimens were drawn into evacuated EDTA-containing blood collection tubes, stored at 4°C and processed within 72 h. After centrifugation of peripheral blood at 1500 rpm for 10 min, the plasma samples were carefully removed from the upper portion of the supernatant. Red blood cell lysis buffer (0.139 M NH_4_Cl, 0.02 M Tris, pH 7.2) was added to the residual blood specimens and mixed for 45 min at room temperature. Following centrifugation at 1500 rpm for 10 min, the supernatant was removed and the residual peripheral blood mononuclear cell (PBMC) pellet was resuspended in PBS buffer.

### Instrument Setup

Before the processing of detection, the concentrations of PBMC samples from patients with rectal cancer (or samples from healthy donors with spiked HT-29 cells) were measured by a hemacytometer and diluted in PBS buffer as 5×10^7^ cells/mL. Then, the 0.5–0.6 mL diluted PBMC samples were introduced into the device by pumping. A syringe pump (PHD 22/2000, HAVARD apparatus, Massachusetts, USA) with a 5 mL syringe for waste collecting was connected to the outlet of the device. The inlet of the device was connected to the blood samples via polymer tubing. The syringe pump was turned on and the pressure adjusted according to the required flow rate. The blood samples were pumped from the inlet into the microfluidic device for CTC capture and the filtered hematologic constituents such as leukocytes were collected into the waste chamber.

### Identification and Enumeration of CTCs by Fluorescence Microscopy

After the process of CTC capture, tumor cells were identified and distinguished from leukocytes based on morphology and differential antigen expression. Tumor cells are epithelial (cytokeratin+/CD45−/DAPI+), whereas leukocytes are nonepithelial (cytokeratin−/CD45+/DAPI+). Fluorescent reagents were pumped into the device and immunofluorescence reaction was done directly in the channels of the device. Firstly, antibodies to CD45 conjugated to FITC (BD Biosciences, USA) were introduced into the device, followed by incubation for 30 min at 0°C. Subsequently, a solution of 0.2% Triton X-100 and antibodies to cytokeratin conjugated to phycoerythrin (C-11, Abcam, UK) were pumped into the device and incubated for 10 min and 1 h, respectively. Then, captured cells were mixed with DAPI solution for 20 min. Finally, captured cells were fixed by a solution of 1% paraformaldehyde for 20 min. After these reactions, the device was flushed with 1 mL PBS to remove excess reagents. Captured cells were identified and enumerated using fluorescence microscopy (Olympus America). CTCs were identified according to the stained color and morphological characteristics such as cell size, shape and nuclear size. The cells that stained cytokeratin+/CD45−/DAPI+ and met the phenotypic morphological characteristics were scored as CTCs. An experienced pathologist who was blind to the clinical results comprehensively evaluated each blood sample for CTC identification.

### CTCs Detection by EpCAM-based Method

The capture efficiency of CTCs was also measured by EpCAM-based method. Detailed method was described as follows: The separation of CTCs was performed by use of immunomagnetic beads coated with epithelial cell-specific EpCAM antibodies (magnetic bead: 4.5 µm diameter; DYNAL, Invitrogen, USA) according to the manufacturer's instruction. The PBMC pellet was resuspended in PBS buffer and mixed with immunomagnetic beads. The cell-beads mixture was incubated for 40 min at 2–8°C with gentle tilting and rotation. The mixture was placed in a magnetic for 5 min and the supernatant was removed carefully. Subsequently, antibodies to CD45 conjugated to FITC (BD Biosciences, USA) were added and mixed with captured cells for 30 min at 0°C. Then cells were washed with a solution of 0.2% Triton X-100 for 10 min and mixed with antibodies to cytokeratin conjugated to phycoerythrin (C-11, Abcam, UK) for 1 h. After that, captured cells were mixed with DAPI solution for 20 min. Finally, cells were fixed by a solution of 1% paraformaldehyde for 20 min. After each processing step, captured cells were washed by PBS buffer to remove excess reagents in a magnetic. The method of identifying and enumerating CTCs was similar with that of microfluidic device, according to the stained color and morphological characteristics such as cell size, shape and nuclear size. The cells that stained cytokeratin+/CD45−/DAPI+ and met the phenotypic morphological characteristics were scored as CTCs. An experienced pathologist who was blind to the clinical results comprehensively evaluated each blood sample for CTC identification.

### Ethics Statement

All procedures of enrollment were performed in compliance with relevant laws and institutional guidelines and relevant institutional committee (Ethics Committee of Fudan University Shanghai Cancer Center) approved our research. All patients and healthy volunteers signed written informed consent prior to enrollment.

### Statistical Analysis

Statistical analyses were performed using SPSS software (version 16.0, SPSS Inc., Chicago, IL). All results were expressed as means ± standard deviations (SD). Capture efficiency was calculated by dividing the number of the captured target cells by the number of total target cells introduced into the device. The purity of cells captured was determined by dividing the number of the captured target cells by the number of the total captured cells. Regression analysis was performed to assess the accuracy of tumor cells detection. A Mann-Whitney U test and Kruskal-Wallis H test were used in cases of 2 independent samples and more than 2 samples, respectively, whereas the comparisons of related measurements were performed using a Wilcoxon signed rank test. Spearman's rank correlation was used to analyze the correlation of capture rate between microfluidic device and EpCAM-based method. All tests were two-sided and were performed at a 5% level of significance.

## Results and Discussion

### The Effect of Channel Height and Flow Rate on CTC Capture Efficiency

In order to optimize the experimental parameters of this device, we spiked HT-29 cells with known numbers into blood samples from healthy donors to measure capture efficiency and cell purity.

**Figure 2:**
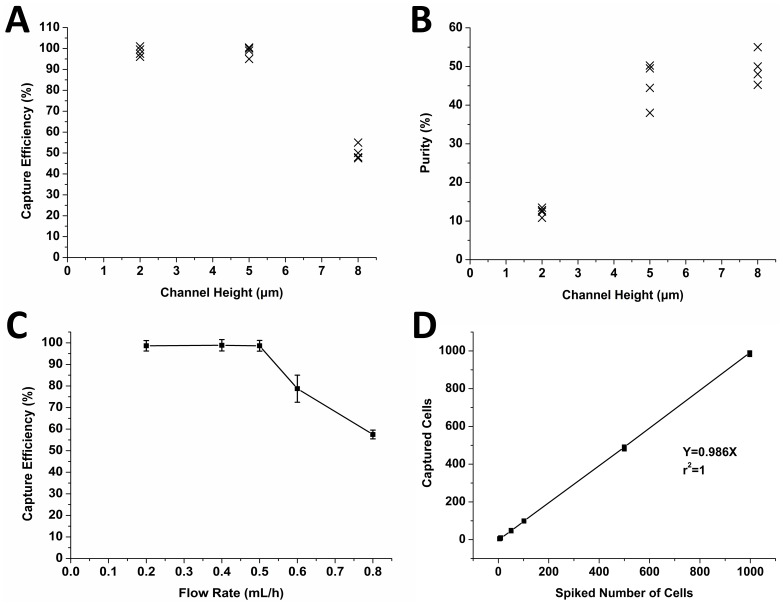
The parameter optimization of the microdevice and recovery rate of tumor cells. A) The relationship between channel height and capture efficiency: The capture efficiency was calculated by dividing the number of the target cells captured by the number of the target cells introduced into the device. B) The relationship between channel height and cell purity: The purity of cells captured was determined by dividing the number of the target cells captured by the number of the total cells captured. The flow rate was 0.5 mL/h for A) and B). C) The relationship between flow rate and capture efficiency: The capture efficiency was calculated by dividing the number of the target cells captured by the number of the target cells introduced into the device. The channel height was 0.5 µm. The error bars represented one standard deviation of 4 repeat experiments. D) Recovery of known numbers of spiked HT-29 cells from whole blood. Regression analysis of observed tumor cells number versus expected tumor cells number produced a slope of 0.986 and a correlation coefficient (R^2^) of 1.

Firstly, we investigated the effects of the filter channel height on capture efficiency and cell purity. As shown in Figure 2a, the capture efficiency of CTCs with 8 µm channel height was obviously lower than that with 2 µm and 5 µm height, while similar higher capture efficiency of 98% could be observed for 2 µm and 5 µm. In addition, the cell purity gradually improved with the increase of the channel height, as demonstrated in Figure 2b. Thus, on the basis of the data in Figure 2a and 2b, we chose an height of 5 µm as the best trade-off between the capture efficiency and cell purity. These results indicated that there was a close relationship between the size of filter cavity and the capture efficiency of CTCs, and some other studies also made similar conclusions: Hosokawa M. et al. [24] utilized a size-selective microcavity array to isolate CTCs and its capture efficiency presented a downward trend with the increase of microcavity size. Sheng W. et al. [25] fabricated an aptamer-enabled microdevice and also indicated that the capture efficiency of CTCs would descend gradually but the purity had a upward trend when the channel depth increased.

On the other hand, we also investigated the relationship between flow rate and CTC capture efficiency. As shown in Figure 2c, at the flow rate ranging from 0.2 and 0.5 mL/h, the capture efficiency did not change obviously. But from the rate above 0.5 mL/h, the capture efficiency declined dramatically. The capture efficiency reduced with the increase of flow rate presumably because of a larger pressure at a higher flow rate. Some other studies [24]–[26] also pointed out that cell capture efficiency was inversely proportional to the flow rate of blood. Therefore, considering the processing time of samples pumped into the device and the capture efficiency, we chose 0.5 mL/h as the best parameter for detection, at which flow rate sufficient throughput could be observed.

Therefore, we used the optimal channel height of 5 µm and flow rate of 0.5 mL/h for CTC detection, and these experimental conditions were used for all subsequent experiments.

### Recovery of Tumor Cell Detection

To determine the sensitivity of tumor cell detection, HT-29 cells at different known numbers, were spiked into 5 ml blood samples from each healthy donor. The numbers of spiked cells were 5, 9, 51, 102, 500 and 998 respectively. Figure 2d showed the expected number of HT-29 cells spiked into the healthy donor samples plotted against the actual number of HT-29 cells observed in the samples. Regression analysis of observed tumor cells number versus expected tumor cells number produced a slope of 0.986 and a correlation coefficient (R^2^) of 1. The mean percentage of HT-29 recovered was 94% and the recovery rates at various cell concentrations were all above 80% (Table 1, Table S1). We also validated the recovery rates of three lung cancer cell lines including A549, SK-MES-1 and H446, all of which were higher than 90% (data not shown).

**Table 1:** Comparison of two method accuracy measured by recovery of HT-29 cells spiked into 5 mL blood of healthy donors.

Expected CTCs count	Microfluidic method				EpCAM-based method			
	Observed CTCs count		% Recovery		Observed CTCs count		% Recovery	
	mean±SD	range	mean±SD	range	mean±SD	range	mean±SD	range
5	4±0	4–5	84±9	80–100	2±1	0–4	36±30	0–80
9	8±1	7–10	93±13	78–111	5±1	4–7	60±13	44–78
51	47±1	46–49	92±2	90–96	41±3	38–46	81±7	75–90
102	99±1	98–99	97±1	96–97	77±8	68–87	75±7	67–85
500	487±3	480–491	97±1	96–98	398±15	380–420	80±3	76–84
998	988±4	980–990	99±0	98–99	699±15	688–720	70±1	69–72

### CTC Detection of Rectal Cancer Patients with Various Stages

**Figure 3:**
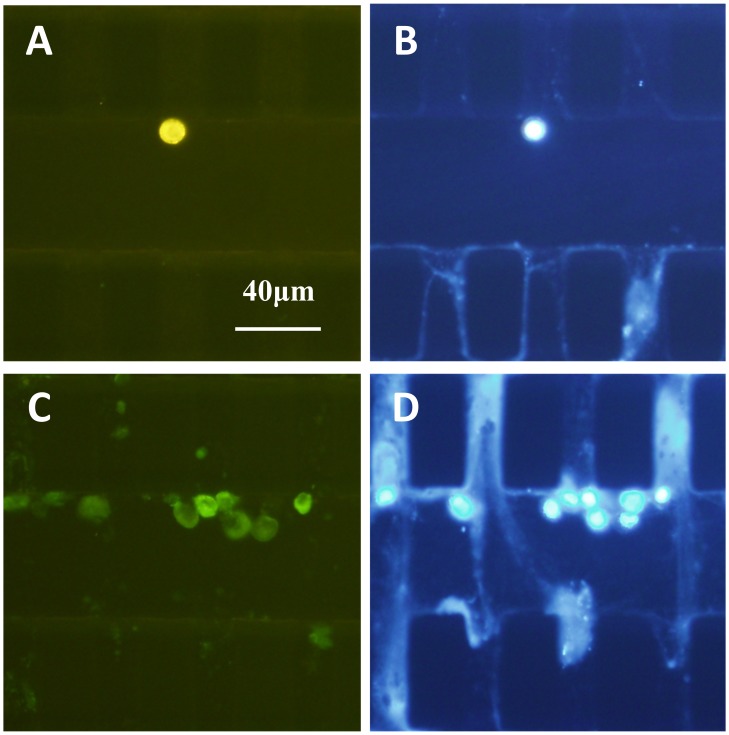
Circulating tumor cells and normal blood cells in one typical patient with rectal cancer. A/B: CTCs staining; C/D: normal blood cells staining. A) positive CK staining in CTCs; B) positive DAPI staining in CTCs; C) positive CD45 staining in normal blood cells; D) positive DAPI staining in normal blood cells.

The CTC counts of 60 rectal cancer patients with different stages and 30 healthy subjects were detected and analyzed in each 5 mL whole blood. Figure 3 demonstrated the captured CTCs of one typical patient with rectal cancer in the microfluidic device.

**Figure 4:**
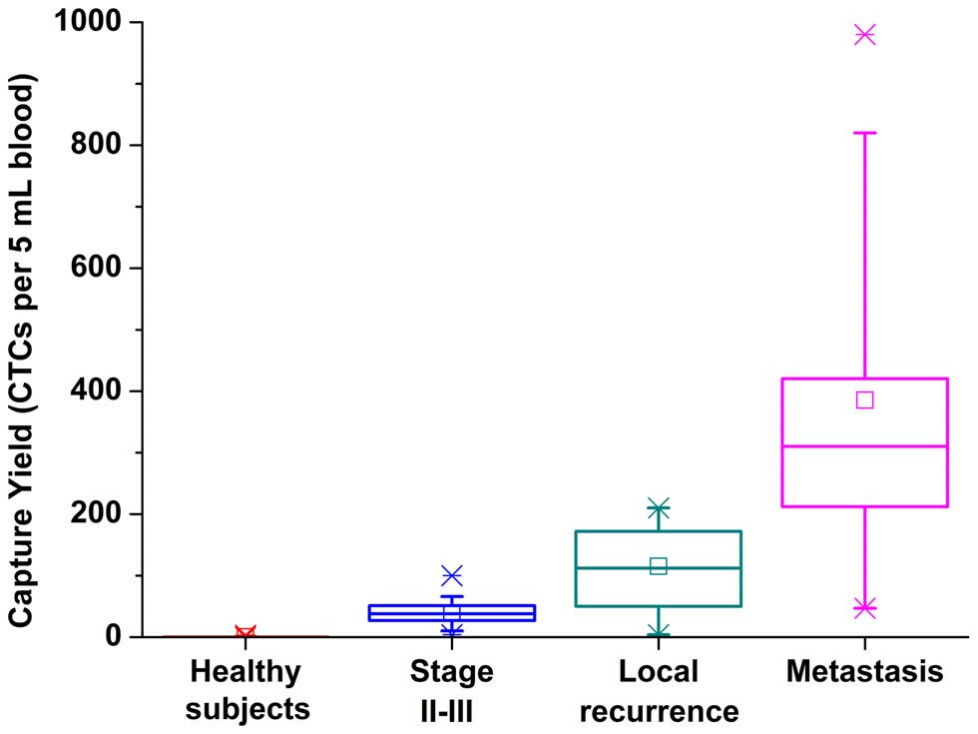
Enumeration of CTCs from patients with rectal cancer. The box plot demonstrates the median, lower and upper quartiles (25th, 75th percentiles). Data points that outside the 10th and 90th percentiles are shown as outliers.

As demonstrated in Figure 4, positive cells could be observed in 3 out of 30 healthy people, but none of them had more than 3 positive cells per sample. These results were similar with those in previous studies indicating that up to three CTCs were detected in a small population of healthy donors or nonmalignant patients [27]–[28]. The reason for such "false positive" results is unknown yet, but presumably because of several reasons as follows: (a) contamination of epithelial cells in blood samples due to some incorrect processing such as inappropriate blood sampling; (b) inaccurate judgment in identification of CTCs by researchers; (c) presence of aberrant hematologic cells which express epithelial antigen in blood samples; (d) potential cancer patients who cannot be early diagnosed by routine clinical techniques.

Although tumor markers such as serum CEA are widely used for the diagnosis and follow-up of patients with gastrointestinal cancers, their lack of sensitivity remains unsolved. Conventional imaging modalities and tumor markers are sometimes inaccurate for cancer diagnosis and monitoring of therapy responses, resulting in a delay in judging disease progression early. In this study, CTCs could be detected in all 60 rectal cancer patients whose CTC counts were all above 3 cells per sample. The CTC counts of rectal cancer patients were significantly higher than those in healthy subjects (167.33±212.76 vs. 0.17±0.59, P<0.05). Additionally, patients with distant metastases had significantly higher CTC levels than patients with Stage II-III or those with local recurrence (385.30±245.86 vs. 39.40±19.23 vs. 115.20±69.15, P<0.05). Furthermore, the CTC counts of local recurrent patients were also significantly higher than those with Stage II-III (P<0.05). These results indicated that CTC quantity correlated reasonably well with the tumor burden and the severity of the patients with different stages. Therefore, CTC might be a promising biomarker to make up the insufficiency of conventional clinical detection modalities. Sastre et al. [29] demonstrated a close relationship between CTC counts and different clinical stages for colorectal cancer patients, which was similar to our results. Cohen et al. [30] indicated that CTC counts before treatment and at subsequent time points were independent prognostic factors for progression-free survival and overall survival. Allen-Mersh et al. [31] showed that CTC counts within 24 h after primary colorectal cancer resection was a strong predictor of colorectal cancer recurrence. The detection of CTCs might also be applied in targeted gene therapy. Yen et al. [32] found that the detection of Kras mutational status in CTCs had potential for clinical application in selecting metastatic colorectal cancer patients most likely to benefit from cetuximab therapy. Thus, the detection of CTCs may have a wide-range application in the clinical settings such as the diagnosis of cancer diseases, monitoring of treatment responses, prognosis evaluation and even the decision-making of individualized therapy. Further analyses including the role of CTCs on prediction and evaluation of cancer patients’ long-term survival using our microfluidic device will be undertaken in the near future.

### Comparison Microfluidic Device vs. EpCAM-based Method

Anti-EpCAM magnetic bead-based method is currently one of the most commonly used technologies for CTC detection [9]–[10]. Therefore, we compared the performance of our microdevice for enumeration of tumor cells against EpCAM-based enrichment method. Firstly, HT-29 cells with known numbers were spiked into blood samples from healthy donors, and then each sample was split into two duplicates, one for our device and another for EpCAM-based method. The recovery rates of these two methods were compared and analyzed. As shown in Table 1, the recovery rates of tumor cells using this microdevice were all above 80% at each concentration of tumor cells, whereas the recovery rates by EpCAM-based method were comparatively lower, ranging from 36% to 81% (P<0.05). In addition, we could not find significant correlation between the recovery rate of microfluidic device and that of EpCAM-based method (Correlation efficient was 0.07, P>0.05.). Subsequently, we also compared the performance of these two methods using patients’ blood samples and the results were demonstrated in Table 2. We collected and processed the blood samples of 60 patients with rectal cancer, and each sample was divided into two pieces for tumor cells detection using these two methods (Table S2). For rectal cancer patients with different stages, the CTC counts detected by microdevice were all significantly higher than those by EpCAM-based method (P<0.05). The number of isolated CTCs ranged from 0 to 100 versus 0 to 12 (microdevice vs. EpCAM-based) per sample for patients with Stage II-III (mean±SD, 39±19 vs. 4±3), 4 to 210 vs. 3 to 31 for recurrent patients (115±69 vs. 12±9) and 47 to 980 vs. 5 to 55 for metastatic patients (385±246 vs. 24±15). For EpCAM-based method, the CTC counts of 38 (63%) patients ranged from 0 to 10, whereas that in only one patient (2%) was more than 50. By contrast, for microdevice, the CTC counts of 3 (5%) patients ranged from 0 to 10 while those in 34 (57%) patients were more than 50. The difference between these two methods was especially obvious for 30 rectal cancer patients with Stage II-III: The positive rates of samples with more than 3 CTCs per 5 mL blood by use of microdevice were 100% (30/30), whereas only 47% (14/30) positive samples could be observed by EpCAM-based method.

**Table 2:** Comparison of two methods in CTCs counts per 5

Cohort		Total No. Of sample	CTCs counts per 5 mL blood (mean±SD)	Range of CTCs per 5 mL blood: No. of samples(%)					Samples with >3 CTCs per 5 mL blood (%)
				0–10	11–50	51–100	101–500	>500	
Microfluidic method	Stage II-III	30	39±19	2 (6)	20 (67)	8 (27)	0 (0)	0 (0)	30 (100)
	Local recurrence	10	115±69	1 (10)	2 (20)	1 (10)	6 (60)	0 (0)	10 (100)
	Metastasis	20	385±246	0 (0)	1 (5)	0 (0)	15 (75)	4 (20)	20 (100)
EpCAM-based method	Stage II-III	30	4±3	28 (93)	2 (7)	0 (0)	0 (0)	0 (0)	14 (47)
	Local recurrence	10	12±9	6 (60)	4 (40)	0 (0)	0 (0)	0 (0)	9 (90)
	Metastasis	20	24±15	4 (20)	15 (75)	1 (5)	0 (0)	0 (0)	20 (100)

EpCAM-magnetic bead based method is currently one of the most prevalent approaches for the isolation of CTCs. However, this method is dependent on the EpCAM expression on the target tumor cells. Although EpCAM antigen can be found in the majority of epithelial tumors, weak or negative EpCAM expression has been indicated, which is probably associated with the phenomenon of EMT [33]–[34]. Many factors such as chemotherapy can probably induce EMT, which can affect the capture efficiency of tumor cells. In our study, in order to minimize the influence of EMT, we utilized a size-based method to detect CTCs instead of EpCAM-dependent one. Hence, theoretically, more tumor cells including both EpCAM-expression positive and negative ones could be captured by our size-based microdevice. This hypothesis is in accordance with our results above: Not only in samples with tumor cells spiked into healthy blood, more CTCs were isolated by our microdevice, but similar results were validated in samples of rectal cancer patients as well. Other previous studies [35]–[36], which compared size-based and EpCAM-based method, reported similar findings and demonstrated that a proportion of cells isolated by size-based approach lacked epithelial characteristics.

On the other hand, we also measured the sizes of captured tumor cells in our study. Firstly, we compared the sizes of HT-29 cells before and after capture using our microfluidic device (Table S1). No significant difference in cell size was found before and after capture (mean ± SD, before capture: 16.49±1.10 µm vs. after capture: 16.49±1.09 µm, P>0.05), which indicated that our microfluidic device could capture HT-29 cells with various sizes. In addition, we also compared the sizes of captured CTCs using our microfluidic device and EpCAM-based method (Table S2), and similar cell size distribution was also found in these two methods (mean ± SD, microfluidic device: 16.42±1.24 µm vs. EpCAM-based method: 16.40±1.66 µm, P>0.05). However, the smallest size of captured CTCs was 10 µm using our microfluidic device. By contrast, by use of EpCAM-based method, a total of 8 tumor cells with size smaller than 10 µm (5–8 µm) were found in 3 out of 60 cancer patients. This result indicated that our microfluidic device might miss some of tumor cells especially the cells with smaller sizes.

Although our microfluidic device showed a better performance than EpCAM-based method in our study, some points should also be addressed. Firstly, these results should be validated with more standard EpCAM-based method e.g. CellSearch. Although we have optimized various experimental parameters and got better CTC capture rate for our EpCAM-based method, our microfluidic device should also be compared with CellSearch in order to verify the results of comparison between our microfluidic device and EpCAM-based method. Secondly, our microfluidic device is a size-based detection system, so it should also be compared with other size-based methods e.g. ISET. We will update these results if these commercially used methods are introduced in our hospital in the future. Thirdly, although our microfluidic device could capture much more CTCs than EpCAM-based method in our study, some CTCs were still missed using our microfluidic device especially some smaller tumor cells. Nevertheless, these smaller tumor cells could be captured by EpCAM-based method, which was independent of cell size. Each kind of detection system has its own features and limitations. Therefore, new techniques combining the advantages of different methods might be more promising in the future.

## Conclusion

We demonstrated a simple and economical size-based microfluidic device to isolate CTCs in patients with rectal cancer. The unique design of microchannel array in the device resulted in the high-performance cell isolation. Although further verification and analyses should be undertaken in the future, this device might provide a robust platform aimed at early cancer diagnosis, monitoring treatment responses and predicting cancer prognosis.

## Supporting Information

Table S1 **Counts and sizes of HT-29 cell lines before and after capture.**
(DOC)

Table S2 **Counts and sizes of CTCs in rectal cancer patients using two different methods.**
(DOC)
