# Supplementary material for: High-Performance Size-Based Microdevice for the Detection Of Circulating Tumor Cells from Peripheral Blood in Rectal Cancer Patients
Source: PLoS One. 2013 Sep 16;8(9):e75865. doi: 10.1371/journal.pone.0075865 (PMC3774665; doi:10.1371/journal.pone.0075865)
Supplement: Table S1 — Counts and sizes of HT-29 cell lines before and after capture. (DOC) [file pone.0075865.s001.doc]

| Sample No. of HT-29 | Counts before capture | Counts after capture | Size before capture | Size after capture |
| --- | --- | --- | --- | --- |
| 1 | 5 | 5 | 16.80±0.84 | 16.80±0.84 |
| 2 | 5 | 4 | 16.80±0.84 | 17.00±0.82 |
| 3 | 5 | 4 | 16.80±0.84 | 17.00±0.82 |
| 4 | 5 | 4 | 16.80±0.84 | 16.75±0.96 |
| 5 | 5 | 4 | 16.80±0.84 | 16.50±0.58 |
| 6 | 9 | 9 | 16.44±0.73 | 16.44±0.73 |
| 7 | 9 | 8 | 16.44±0.73 | 16.50±0.76 |
| 8 | 9 | 9 | 16.44±0.73 | 16.56±0.88 |
| 9 | 9 | 8 | 16.44±0.73 | 16.75±1.17 |
| 10 | 9 | 7 | 16.44±0.73 | 16.57±0.79 |
| 11 | 51 | 47 | 16.49±0.95 | 16.64±0.99 |
| 12 | 51 | 46 | 16.49±0.95 | 16.59±0.93 |
| 13 | 51 | 49 | 16.49±0.95 | 16.55±0.96 |
| 14 | 51 | 46 | 16.49±0.95 | 16.48±0.94 |
| 15 | 51 | 47 | 16.49±0.95 | 16.38±0.90 |
| 16 | 102 | 98 | 16.59±0.96 | 16.74±0.79 |
| 17 | 102 | 99 | 16.59±0.96 | 16.56±0.88 |
| 18 | 102 | 99 | 16.59±0.96 | 16.52±0.87 |
| 19 | 102 | 98 | 16.59±0.96 | 16.63±0.94 |
| 20 | 102 | 99 | 16.59±0.96 | 16.61±0.97 |
| 21 | 500 | 490 | 16.26±1.07 | 16.22±1.05 |
| 22 | 500 | 480 | 16.26±1.07 | 16.35±1.04 |
| 23 | 500 | 487 | 16.26±1.07 | 16.26±1.06 |
| 24 | 500 | 487 | 16.26±1.07 | 16.23±1.03 |
| 25 | 500 | 491 | 16.26±1.07 | 16.32±1.00 |
| 26 | 998 | 990 | 16.59±1.13 | 16.56±1.13 |
| 27 | 998 | 990 | 16.59±1.13 | 16.57±1.14 |
| 28 | 998 | 980 | 16.59±1.13 | 16.58±1.11 |
| 29 | 998 | 990 | 16.59±1.13 | 16.59±1.12 |
| 30 | 998 | 990 | 16.59±1.13 | 16.61±1.10 |
