# Supplementary material for: High-Performance Size-Based Microdevice for the Detection Of Circulating Tumor Cells from Peripheral Blood in Rectal Cancer Patients
Source: PLoS One. 2013 Sep 16;8(9):e75865. doi: 10.1371/journal.pone.0075865 (PMC3774665; doi:10.1371/journal.pone.0075865)
Supplement: Table S2 — Counts and sizes of CTCs in rectal cancer patients using two different methods. (DOC) [file pone.0075865.s002.doc]

| Patient No. | Counts (microdevice) | Counts (EpCAM method) | Cell size (microdevice) | Cell size (EpCAM method) |
| --- | --- | --- | --- | --- |
| 1 | 100 | 3 | 16.15±1.21 | 16.33±1.16 |
| 2 | 30 | 5 | 17.03±0.81 | 17.00±1.00 |
| 3 | 47 | 2 | 16.89±1.26 | 17.50±0.71 |
| 4 | 56 | 8 | 17.52±1.03 | 17.50±1.31 |
| 5 | 37 | 1 | 16.35±0.92 | 17.00±0.00 |
| 6 | 27 | 10 | 16.96±0.81 | 16.80±0.63 |
| 7 | 42 | 4 | 16.90±0.76 | 17.00±0.82 |
| 8 | 33 | 5 | 16.67±0.60 | 16.80±0.45 |
| 9 | 66 | 6 | 16.80±0.75 | 16.67±0.82 |
| 10 | 56 | 3 | 16.45±1.11 | 17.00±2.00 |
| 11 | 47 | 1 | 16.51±0.89 | 17.00±0.00 |
| 12 | 38 | 6 | 16.68±0.81 | 16.83±1.33 |
| 13 | 42 | 3 | 16.60±1.04 | 15.67±0.58 |
| 14 | 51 | 1 | 16.98±0.32 | 17.00±0.00 |
| 15 | 47 | 4 | 17.15±0.82 | 17.00±0.00 |
| 16 | 52 | 12 | 17.04±0.77 | 17.17±0.72 |
| 17 | 4 | 6 | 17.00±1.16 | 13.67±5.28 |
| 18 | 17 | 0 | 17.18±0.39 | --- |
| 19 | 23 | 0 | 16.30±0.64 | --- |
| 20 | 47 | 4 | 17.62±1.13 | 18.00±1.16 |
| 21 | 37 | 2 | 17.11±0.32 | 17.00±0.00 |
| 22 | 15 | 0 | 16.33±0.49 | --- |
| 23 | 12 | 0 | 16.42±0.52 | --- |
| 24 | 53 | 5 | 17.49±0.51 | 17.60±0.55 |
| 25 | 49 | 3 | 18.16±1.20 | 18.33±0.58 |
| 26 | 34 | 11 | 16.56±0.50 | 16.82±0.41 |
| 27 | 51 | 4 | 16.43±1.01 | 16.75±1.26 |
| 28 | 10 | 0 | 15.80±0.42 | --- |
| 29 | 36 | 3 | 15.86±0.87 | 16.33±0.58 |
| 30 | 23 | 0 | 17.09±0.29 | --- |
| 31 | 172 | 23 | 16.59±1.17 | 16.70±2.44 |
| 32 | 50 | 3 | 15.70±0.54 | 16.33±1.16 |
| 33 | 210 | 31 | 16.67±1.01 | 16.13±2.22 |
| 34 | 4 | 6 | 15.25±0.96 | 12.83±3.82 |
| 35 | 177 | 8 | 16.58±1.17 | 16.50±1.60 |
| 36 | 170 | 12 | 17.97±0.76 | 17.83±0.94 |
| 37 | 40 | 7 | 15.60±0.67 | 15.86±0.69 |
| 38 | 112 | 12 | 17.44±1.01 | 17.58±1.08 |
| 39 | 137 | 8 | 16.94±1.15 | 16.50±1.07 |
| 40 | 80 | 6 | 16.99±0.68 | 16.67±0.52 |
| 41 | 980 | 40 | 14.48±0.94 | 14.48±2.80 |
| 42 | 330 | 16 | 16.77±0.97 | 16.62±1.03 |
| 43 | 780 | 33 | 17.01±0.81 | 16.64±0.99 |
| 44 | 120 | 15 | 16.35±0.73 | 16.40±0.74 |
| 45 | 460 | 50 | 15.85±1.16 | 15.54±1.58 |
| 46 | 257 | 15 | 16.36±0.85 | 16.60±1.35 |
| 47 | 417 | 25 | 16.35±1.30 | 16.24±1.30 |
| 48 | 310 | 40 | 16.82±0.98 | 16.68±1.25 |
| 49 | 210 | 10 | 16.93±1.02 | 16.80±1.40 |
| 50 | 47 | 15 | 14.49±1.68 | 14.80±1.42 |
| 51 | 680 | 55 | 17.42±0.88 | 17.25±0.89 |
| 52 | 420 | 5 | 16.21±0.64 | 16.40±1.34 |
| 53 | 190 | 25 | 17.12±0.80 | 17.12±0.73 |
| 54 | 300 | 20 | 16.16±0.87 | 16.30±1.13 |
| 55 | 232 | 8 | 16.01±0.50 | 16.12±0.64 |
| 56 | 212 | 13 | 16.61±0.67 | 16.69±0.86 |
| 57 | 302 | 19 | 16.34±0.48 | 16.53±0.51 |
| 58 | 820 | 47 | 16.07±1.25 | 16.36±0.90 |
| 59 | 310 | 10 | 16.75±0.52 | 16.40±0.52 |
| 60 | 329 | 15 | 16.62±0.76 | 16.47±0.83 |
